# Lysosomal Sequestration Impairs the Activity of the Preclinical FGFR Inhibitor PD173074

**DOI:** 10.3390/cells7120259

**Published:** 2018-12-08

**Authors:** Bernhard Englinger, Sebastian Kallus, Julia Senkiv, Anna Laemmerer, Patrick Moser, Lisa Gabler, Diana Groza, Christian R. Kowol, Petra Heffeter, Michael Grusch, Walter Berger

**Affiliations:** 1 Institute of Cancer Research and Comprehensive Cancer Center, Department of Medicine I, Medical University of Vienna, A-1090 Vienna, Austria; bernhard.englinger@meduniwien.ac.at (B.E.); yu.senkiv@gmail.com (J.S.); anna.laemmerer@gmx.net (A.L.); patrick.moser95@gmx.at (P.M.); lisa.gabler@meduniwien.ac.at (L.G.); diana.groza@meduniwien.ac.at (D.G.); petra.heffeter@meduniwien.ac.at (P.H.); michael.grusch@meduniwien.ac.at (M.G.); 2 Institute of Inorganic Chemistry, Faculty of Chemistry, University of Vienna, A-1090 Vienna, Austria; sebastian.kallus@univie.ac.at (S.K.); christian.kowol@univie.ac.at (C.R.K.); 3 Research Cluster "Translational Cancer Therapy Research", A-1090 Vienna, Austria; 4 Institute of Cell Biology, National Academy of Sciences of Ukraine, 79005 Lviv, Ukraine

**Keywords:** cancer, drug sequestration, fibroblast growth factor receptor, fluorescence, lysosomes, TKI

## Abstract

Knowledge of intracellular pharmacokinetics of anticancer agents is imperative for understanding drug efficacy as well as intrinsic and acquired cellular resistance mechanisms. However, the factors driving subcellular drug distribution are complex and poorly understood. Here, we describe for the first time the intrinsic fluorescence properties of the fibroblast growth factor receptor inhibitor PD1703074 as well as utilization of this physicochemical feature to investigate intracellular accumulation and compartmentalization of this compound in human lung cancer cells. Cell-free PD173074 fluorescence, intracellular accumulation and distribution were investigated using analytical chemistry and molecular biology approaches. Analyses on a subcellular scale revealed selective drug accumulation in lysosomes. Coincubation with inhibitors of lysosomal acidification strongly enhanced PD173074-mediated fibroblast growth factor receptor (FGFR) inhibition and cytotoxicity. In conclusion, intrinsic fluorescence enables analysis of molecular factors influencing intracellular pharmacokinetics of PD173074. Lysosome-alkalinizing agents might represent candidates for rational combination treatment, preventing cancer cell-intrinsic PD173074 resistance based on lysosomal trapping.

## 1. Introduction

Clinical safety and efficacy are the two most important determinants for the successful development of pharmacological agents e.g., for cancer therapy. Consequently, the pharmacokinetic and pharmacodynamic properties of novel compounds need to be carefully evaluated. On a systemic level, determination of parameters such as drug plasma half-life, organ distribution as well as metabolization is the basis for detailed (pre-)clinical characterization and successful application of cytotoxic chemotherapeutics as well as modern targeted anticancer agents. The behavior of such compounds within cancer cells is another important determinant of therapeutic efficacy and safety but is, in many cases, insufficiently understood. Hence, there is a need for more detailed knowledge on subcellular drug distribution in order to identify and combat intracellular factors limiting drug efficacy.

FGFR are receptor tyrosine kinases that play vital roles in physiological processes such as tissue development and homeostasis [1,2]. In the last few decades, oncogenic FGFR alterations—including genetic translocation, amplification or mutation—have been identified in multiple cancer types including lung cancer [3,4]. Consequently, aberrant FGFR signaling has emerged as a promising target for anticancer therapy [5,6,7,8]. An array of compounds inhibiting this signaling module has been designed including small molecule tyrosine kinase inhibitors as well as monoclonal antibodies. The latter target either the extracellular receptor domain or act as FGF ligand traps [3]. Thus far, clinical evaluation of these strategies has culminated in the approval of the multi-kinase inhibitor nintedanib for second-line treatment of non-small cell lung adenocarcinoma in Europe [5,9]. Due to the relative novelty of FGFR inhibitors in clinical oncology, knowledge on resistance mechanisms against these agents is scarce [10]. Preclinical data have indicated FGFR gatekeeper mutations [11] or oncogenic bypass by induction of surrogate kinases [12,13] as limiting factors of FGFR-targeted therapeutics.

PD173074 is a small molecule pan-FGFR tyrosine kinase inhibitor that exerts strong anticancer activity in preclinical models of FGFR-driven cancer types [5,14,15]. Severe toxic events have hampered successful translation of this compound into the clinics. Importantly, subcellular kinetics and compartmentalization of PD173074 are widely unknown. The intrinsic fluorescence emitted by several compounds constitutes a powerful asset for their detection at subcellular level. To date, fluorescence activity of only few compounds has been described, including classical chemotherapeutic drugs as well as modern targeted agents such as doxorubicin or gefitinib, respectively [16,17,18,19,20]. Despite this limited number, intrinsic fluorescence has led to significant insights as to how anticancer compounds are sequestered to distinct intracellular organelles and how affects their pharmacologic effects such as drug efficacy, but potentially also toxicity [21,22,23].

In the present study, we demonstrate the intrinsic, label-free fluorescence properties of PD173074. This feature enabled detection of this agent at subcellular level in FGFR1-amplified lung cancer cells in live and fixed cell conditions and revealed lysosomal sequestration to limit its cytotoxic potential. Accordingly, combination treatment with agents targeting lysosomes resulted in synergistic anti-proliferative effects.

## 2. Material and methods

### 2.1. Drugs and Chemicals

PD173074 was obtained from Selleckchem (Munich, Germany), LysoTracker Red^®^ from Thermo Fisher Scientific (Waltham, MA, USA). Bafilomycin A1, chloroquine, and chlorpromazine (CPZ) were purchased from Sigma (St. Louis, MO, USA).

### 2.2. Cell Culture

The human non-small cell lung cancer cell lines NCI-H1703 (CRL-5889) and NCI-H520 (HTB-182), as well as the human, SV-40 large T-antigen-immortalized, broncho-epithelial cell line BEAS-2B (CRL-9609) were obtained from American Type Culture Collection (Manassas, VA, USA). The human large-cell lung carcinoma cell line VL-2 was established from surgical specimen at the Institute of Cancer Research Vienna (EK Nr. 904/2009) [24]. Cells were cultured at 37 °C and 5% CO_2_ in RPMI-1640 containing 10% fetal calf serum (FCS, PAA, Linz, Austria). Cell authentication was performed by array comparative genomic hybridization (aCGH). *Mycoplasma* contamination (Mycoplasma Stain kit, Sigma) was monitored on a regular basis.

### 2.3. Fluorescence Spectroscopy

Three-dimensional fluorescence spectra were obtained using a FluoroMax^®^-4 spectrofluorometer (Horiba, Kyoto, Japan). Data were processed by FluorEssence v3.5 software (Horiba, Kyoto, Japan). Stock solutions of PD173074, chloroquine, and bafilomycin A1 were prepared in dimethylsulfoxide (DMSO) and further diluted with phosphate-buffered saline (PBS) (pH 7.4) or with citrate buffer (pH 4/5/6) to indicated concentrations (final DMSO concentration 1%). Fluorescence spectra were recorded at excitation wavelengths between 220 nm and 420 nm while the emission was within the range of 240–700 nm, with 5 nm excitation and emission slit widths.

### 2.4. RNA Isolation and Quantitative Real-Time PCR (qPCR)

Total RNA was isolated from cell lysates using Trizol reagent (Life Technologies, Carlsbad, CA, USA). cDNA was generated using MMLV reverse transcriptase (Thermo Fisher Scientific). PCR was perfomed using the GoTaq protocol (Promega, Madison, WI, USA) and the following primers: FGFR1 sense: 5′-CCTCTTCTGGGCTGTGCT-3′, *FGFR1* antisense: 5′-CGGGCATACGGTTTGGTT-3′, *ACTB* sense: 5′-GGATGCAGAAGGAGATCACTG-3′, *ACTB* antisense: 5′-CGATCCACACGGAGTACTTG-3′. *ACTB* served as internal control. *FGFR1* expression levels are depicted as difference to *ACTB* cycle thresholds (ΔC_t_) of respective cell lines.

### 2.5. Flow Cytometry

5 × 10^5^ cells were resuspended in serum-free RPMI supplemented with 4-(2-hydroxyethyl)piperazine-1-ethanesulfonic acid (HEPES, 15 mM, Sigma) and 4-morpholine-propanesulfonic acid (MOPS, 2.09 mg/mL, Sigma) and were treated with indicated PD173074 concentrations. Intracellular compound fluorescence in the presence or absence of 1 µM bafilomycin A1 (cotreated for 1 h) or 20 µM CPZ (pretreated for 1 h) was determined on a LSRFortessa flow cytometer (BD Biosciences, East Rutherford, NJ, USA), using 355, 405, 488 and 640 nm laser excitation wavelengths and DAPI (450/40 nm), Horizon V450 (450/40 nm), FITC (530/30 nm) and APC (660/20 nm) bandpass emission filters, respectively. Data were analyzed using Flowing Software (version 2.5.1, University of Turku, Turku, Finland) and fluorescence intensities are plotted as arbitrary units (a.u.).

### 2.6. Live Cell Microscopy

Cells (5 × 10^4^) were plated in 8-well chamber slides (Ibidi, Martinsried, Germany) and allowed to adhere overnight. Cells were treated with indicated concentrations of PD173074 and imaged on a time-lapse microscope (Visitron Systems, Puchheim, Germany) in the presence or absence of 500 nM LysoTracker Red^®^ with a 40× immersion oil lens using DIC and DAPI channels (395/25 nm excitation and 460/50 nm bandpass emission filter for DAPI) (VisiView software, Visitron Systems). For combination experiments, cells were preincubated with 10 µM PD173073 for 1 h and then treated with 100 µM chloroquine or 1 µM bafilomycin A1 and imaged at the indicated time points. Alternatively, cells were preincubated for 1 h with 1 µM Bafilomycin A1, followed by incubation with 10 µM PD173074 and imaging at the indicated time points.

### 2.7. Confocal Fluorescence Microscopy

Cells (5 × 10^3^) were plated in 8-well chamber slides (Ibidi). When adherent, cells were treated simultaneously with 10 µM PD173074 and 500 nM LysoTracker Red^®^ (Thermo Fisher Scientific) for 1 h. Cells were fixed with 4% paraformaldehyde (PFA) for 20 min. Images were acquired on a confocal laser scanning microscope (LSM700, Zeiss, Jena, Germany) and a 63× immersion oil objective and Zen2010 software (Zeiss) using 405 nm (PD173074) or 555 nm (LysoTracker Red^®^) laser lines and 420 nm and 559 nm longpass emission filters, respectively. Colocalization was calculated using ImageJ thresholded Manders‘ Co-localization Coefficient (MCC), where 0 defines no and 1 a complete co-localization [25]. Ten to twenty individual cells were analyzed individually from at least three independent micrographs. Significance of pixel intensity overlaps was evaluated using ImageJ (1.48v, Bethesda, MD, USA) Costes Colocalization Test [26]. According to this algorithm, colocalization significance is reached above the significant threshold of 0.95.

### 2.8. Western Blot Analysis

Cells were seeded at a density of 5 × 10^5^ in 6-well plates and allowed to adhere overnight. Cells were lysed directly or pretreated 30 min 50 µM or 100 µM chloroquine, followed by coincubation with PD173074 at concentrations and durations as indicated in corresponding figures or figure legends. Sodium dodecyl sulfate—polyacrylamide gel electrophoresis (SDS-PAGE) was performed to separate whole-cell protein extracts. Proteins were transferred onto polyvinylidene difluoride membranes (PVDF, Thermo Fisher Scientific). Anti-FGFR1 (D8E4), anti-p44/42 MAPK (Erk1/2) (137F5), anti-phospho-p44/42 MAPK (Erk1/2) (Thr202/Tyr204) (D13.14.4E), anti-AKT (pan) (C67E7), anti-phospho-AKT (Ser473) (D9E), anti-PLCγ1 (D9H10), and anti-phospho-PLCγ1 (Tyr783) (D6M9S) antibodies were purchased from Cell Signaling Technology (Danvers, MA, USA). Anti-ß-actin (AC-15) was obtained from Sigma. Horseradish peroxidase (HRP)-coupled secondary antibodies were purchased from Santa Cruz Biotech (Dallas, TX, USA).

### 2.9. Cell Viability Assay

Cells were seeded at a density of 3 × 10^3^ in 96-well plates and allowed to adhere overnight. Cells were exposed to increasing concentrations of PD173074 in combination with chloroquine or bafilomycin A1 and cell viability was determined using 3-(4,5-dimethylthiazol-2-yl)-2,5-diphenyltetrazolium bromide (MTT) vitality assay (EZ4U, Biomedica, Vienna, Austria). GraphPad Prism 5 software (5v., La Jolla, CA, USA) was used to generate dose-response curves. IC_50_ values were calculated, indicating PD173074 concentrations yielding a 50% cell viability reduction compared to untreated controls. Synergism of drug combinations was evaluated using Calcu Syn 2.11 software (Biosoft, Ferguson, MO, USA) according to Chou-Talalay and expressed as combination index (CI) [27]. CI values below 0.9, between 0.9–1.2 or above 1.2 indicated synergism, additivity, and antagonism, respectively.

### 2.10. Statistical Analysis

Data were analyzed using GraphPad Prism Prism5 software. Values are given as mean ± standard deviation (SD). If not stated otherwise in the materials and methods section or in the figure legends, one representative result, performed in triplicates for each data point, is depicted out of at least three independent experiments. Statistical significance was evaluated using student’s *t*-test or two-way analysis of variance (ANOVA) and Bonferroni post-test. *p*-values below 0.05. * *p* < 0.05; ** *p* < 0.01; *** *p* < 0.001 were considered statistically significant.

## 3. Results

### 3.1. PD173074 Emits Fluorescence Under Cell-Free Conditions and in Treated Lung Cancer Cells

**Figure 1:**
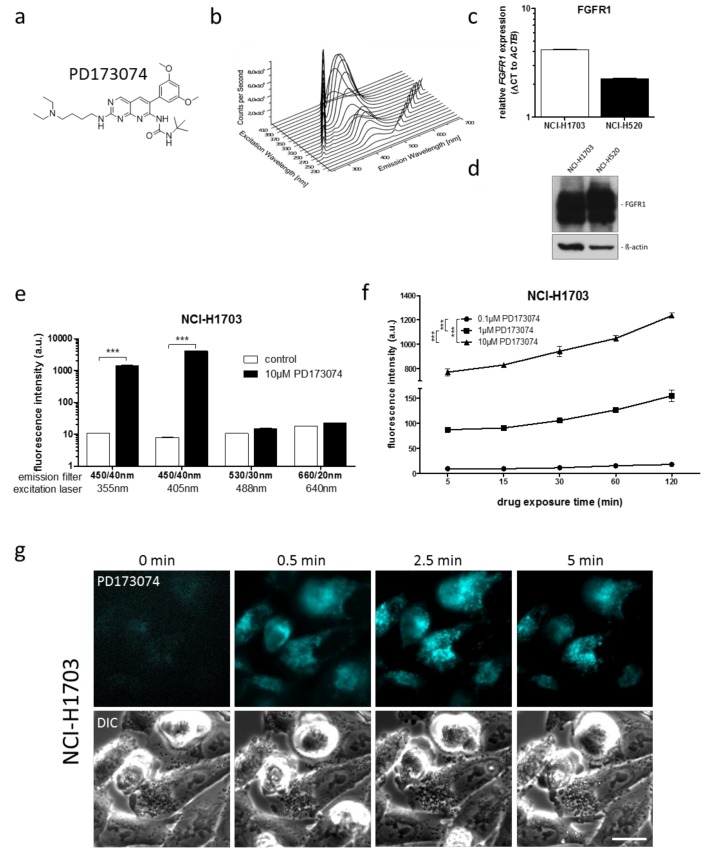
**Intrinsic PD173074 fluorescence activity enables its intracellular detection in vitro.** (**a**) Chemical structure of PD173074. (**b**) A 3-dimensional, full-range excitation-emission spectrum was generated by fluorescence spectroscopy to analyze cell-free fluorescence properties of 15 µM PD173074 at pH 7.4 (diluted in 1% DMSO/PBS). Excitation wavelengths ranged from 220 nm to 420 nm, emission spectra were recorded from 250 nm to 700 nm. Diagonal ridges indicate Raleigh scatters of first and second order. (**c**) FGFR1 mRNA levels in NCI-H1703 and NCI-H520 cells were determined by qPCR. In each cell line, mRNA levels are given as ΔC_t_ to respective ACTB expression. (**d**) FGFR1 protein expression in NCI-H1703 and NCI-H520 cells was determined by Western blot analysis. ß-actin served as loading control. (**e**) Intracellular fluorescence of NCI-H1703 cells, treated for 1 h with 10 µM PD173074 was determined by flow cytometry. Fluorescence emission was detected using DAPI (450/40 nm), Horizon V450 (450/40 nm), FITC (530/30 nm) and APC (660/20 nm) emission channels for the 355 nm, 405 nm, 488 nm, and 640 nm lasers, respectively. *** *p* < 0.001, student’s *ml*-test. (**f**) Accumulation of indicated PD173074 concentrations in NCI-H1703 cells was measured over time by flow cytometry using the 405 nm laser and the Horizon V450 (450/40 nm) emission filter. *** *p* < 0.001, two-way ANOVA, Bonferroni post-test. (**g**) Intracellular PD173074 accumulation in NCI-H170 cells, treated with 10 µM of the drug, was analyzed by live cell microscopy using the DAPI channel. The scale bar indicates 10 µm.

We initially aimed at characterizing the intrinsic fluorescence properties of PD173074 (Figure 1a). Cell-free, three-dimensional (3D) excitation-emission fluorescence spectroscopy revealed distinct fluorescence activity of PD173074, with an emission maximum at 426 nm at an excitation of 370 nm at pH 7.4 (Figure 1b). In order to investigate the intracellular behavior of the drug, the FGFR1-amplified non-small cell lung cancer cell lines NCI-H1703 and NCI-H520 were used. These cell lines exhibit high FGFR1 expression levels (Figure 1C,D) and are sensitive to pharmacological FGFR inhibition [20,28,29]. Flow cytometry analyses revealed that PD173074 was detectable in both cell models when excited with the 355 nm and 405 nm lasers using a 450/40 bandpass emission filter (Figure 1E; Table 1; Figure S1A).

Furthermore, flow cytometry analysis revealed dose- and time-dependently increasing fluorescence intensities in treated cells (Figure 1F; Figure S1B). Cellular PD173074 uptake and subcellular distribution was further confirmed by fluorescence microscopy, where live cell microscopic imaging across a series of time points revealed rapid intracellular PD173074 accumulation already 30 sec after drug exposure (Figure 1G). Interestingly, a dynamic shift in drug distribution over time was apparent, as after 2.5 min, a clearly focal intracellular fluorescence pattern was observed, suggesting distinct subcellular drug compartmentalization. In summary, the intrinsic fluorescence activity of PD173074 enabled sensitive intracellular drug visualization as well as determination of drug uptake kinetics in various live cell approaches.

### 3.2. PD173074 Localizes Selectively to Lysosomes of FGFR1-Driven Lung Cancer Cells

**Figure 2:**
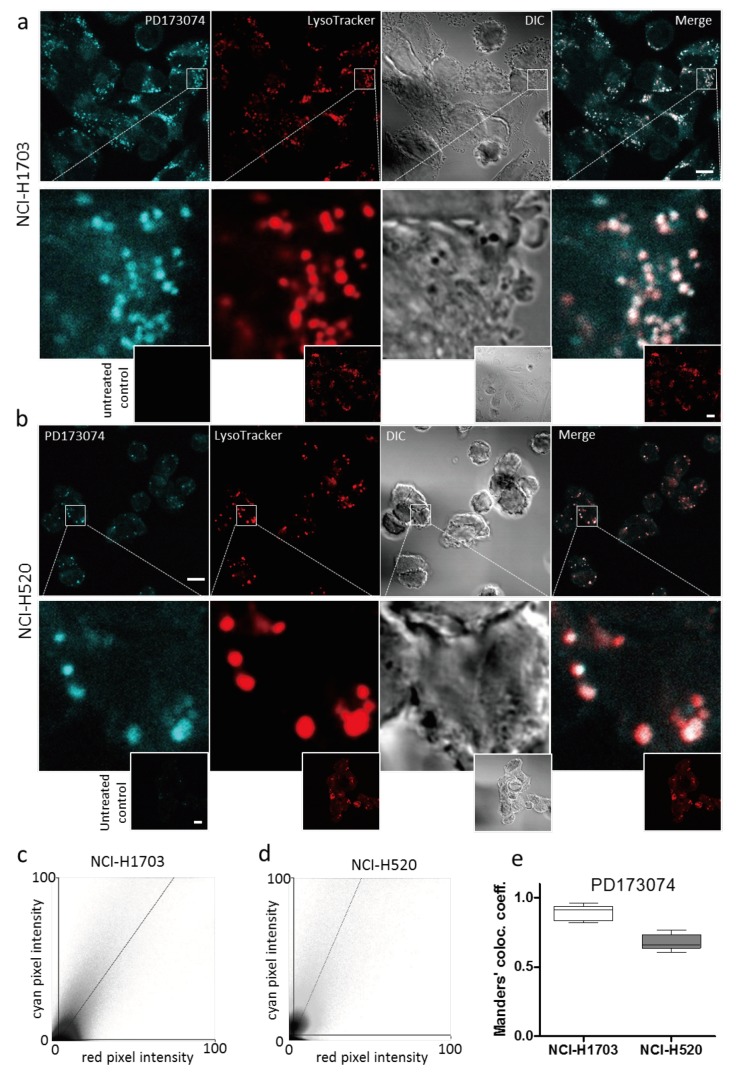
**PD173074 selectively accumulates in lysosomes.** (**a**,**b**) Intracellular PD173074 distribution in NCI-H1703 (**a**) and NCI-H520 (**b**) cells, exposed to 10 µM of the drug for 1 h was investigated by confocal microscopy using the DAPI channel. Lysosomes were stained with LysoTracker Red^®^. The scale bar indicates 10 µm. (**c**,**d**) Scatter plots showing correlations of PD173074/LysoTracker Red^®^ pixel intensities in NCI-H1703 (**c**) and NCI-H520 (**d**) cells, derived from micrographs depicted in Figure 2a,b, respectively. (**e**) PD173074/Lysotracker Red^®^ colocalization was evaluated by determining thresholded MCC from the fluorescence signals in NCI-H1703 and NCI-H520 cells.

As mentioned in Section 3.1 (compare Figure 1g), live cell microscopic imaging of PD173074 suggested intracellular drug compartmentalization. Indeed, confocal microscopy of NCI-H1703 and NCI-H520 cells simultaneously labelled with the lysosomal dye LysoTracker^®^ Red indicated a marked spatial overlap with PD173074 (Figure 2A,B). Fluorescence intensity correlations calculated from cyan/red pixel intensity scatter plots by the thresholded MCC approach yielded high colocalization coefficients (0.89 and 0.68 for NCI-H1703 and NCI-H520 cells, respectively; Figure 2c–e). To exclude that PD173074 colocalization with LysoTracker^®^ is not merely a reflection of a spectral shift or increased fluorescence activity of the compound in the acidic environment of the lysosomal lumen, we again performed 3D fluorescence spectroscopy, lowering the pH values. This analysis yielded, besides an unaltered excitation/emission spectrum, no detectable increase in PD173074 fluorescence intensity at acidic pH as compared to neutral pH conditions (Figure S1C). This indicates selective localization of PD173074 to the lysosomal cell compartment. Interestingly, lysosome selectivity of PD173074 proved to be independent of FGFR expression, as well as of the cellular transformation state. The inhibitor also accumulated in lysosomes of FGFR1-negative VL-2 lung cancer cells [24], as well as of the broncho-epithelial cell line BEAS-2B (Figure S1D). In addition, our experimental data are substantiated by the fact that other anticancer agents have been published to be sequestered by lysosomes, including nintedanib, imatinib and sunitinib [19,20,30]. This lysosomotropism is mediated by the lipophilic and weakly basic properties of these compounds, enabling passive diffusion through lipid bilayers and subsequent protonation-based trapping in the lysosomal lumen [21]. Based on our experimental data pointing to PD173074 selectivity for lysosomes, we additionally aimed at modelling subcellular distribution of this drug using mathematical algorithms [31]. Besides cellular factors such as organelle volumes, pH or electric membrane potential, these modelling approaches take into account various physicochemical properties of the investigated compounds per se, including molecular weight, acid dissociation constant (pKa, i.e., the propensity for protonation at a certain pH) and partition coefficient (logP, i.e., lipophilicity). Using publically available data for these properties from ChEMBL (logP at pH 7.4 = 5.16; acidic pKa = 10.12; www.ebi.ac.uk/chembl/; accession date August 15, 2018, 12.15 PM) or PubChem databases (logP = 4.5; https://pubchem.ncbi.nlm.nih.gov/; accession date: August 15, 2018, 12.15 PM) predicted selective PD173074 accumulation in lysosomes (10.1-fold and 9.6-fold over cytoplasm, respectively; data not shown). These in silico predictions clearly support our experimental findings and suggest lysosomotropism of PD173074 based on its lipophilic and weak base properties.

It is commonly assumed that lipophilic, weakly basic pharmacological agents freely diffuse across cellular biomembranes. Nevertheless, we were interested in whether PD173074 uptake and lysosomal localization may—at least partially—be a consequence of endocytic processes such as (receptor-mediated) endocytosis. We, thus, pre-incubated NCI-H1703 cells with CPZ, a chemical inhibitor of clathrin-mediated endocytosis via depletion of clathrin and AP2 from the plasma membrane [32]. Flow cytometric analysis revealed that cell pretreatment with CPZ had only a minor effect on PD173074 uptake (Figure S1E). This argues against this pathway as a major player in the lysosomal accumulation of PD173074. Moreover, we found localization of the FGFR inhibitor in lysosomes to remain stable over an extended time course. Illustratively, washout experiments in which a 1 h incubation of NCI-H1703 cells with increasing drug concentrations was followed by recovery in drug-free medium, showed that PD173074 still compartmentalized to lysosomes after 120 h in a dose-dependent manner (Figure S2).

### 3.3. Luminal alkalinization Abrogates Lysosomal PD173074 Sequestration

**Figure 3:**
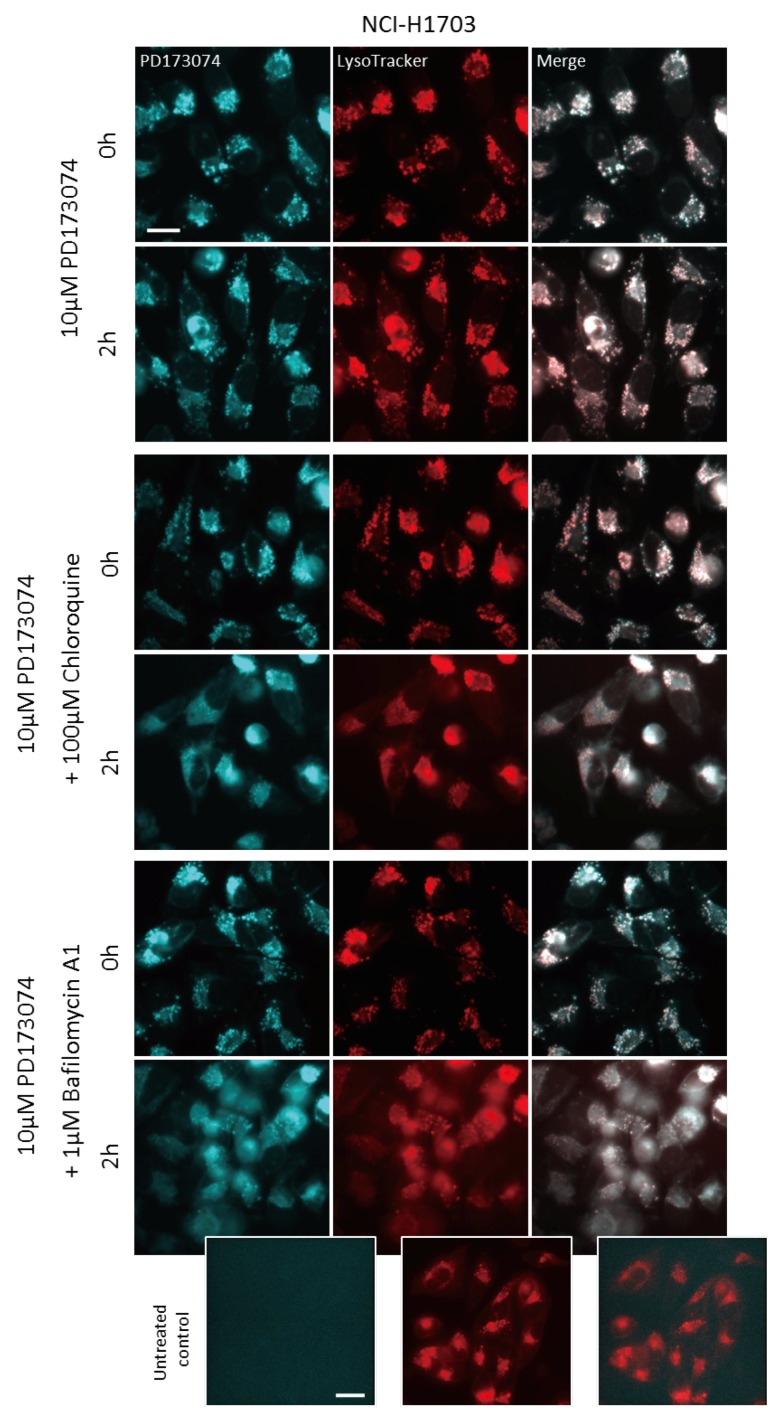
**Luminal alkalinization reduces lysosomal PD173074 accumulation.** Effect of 2 h coincubation with 100 µM chloroquine or with 1 µM bafilomycin A1 on subcellular distribution of PD173074 (10 µM) in NCI-H1703 cells was analyzed by live cell microscopy. LysoTracker Red^®^ was used to stain lysosomes. The scale bar indicates 10 µm.

**Figure 4:**
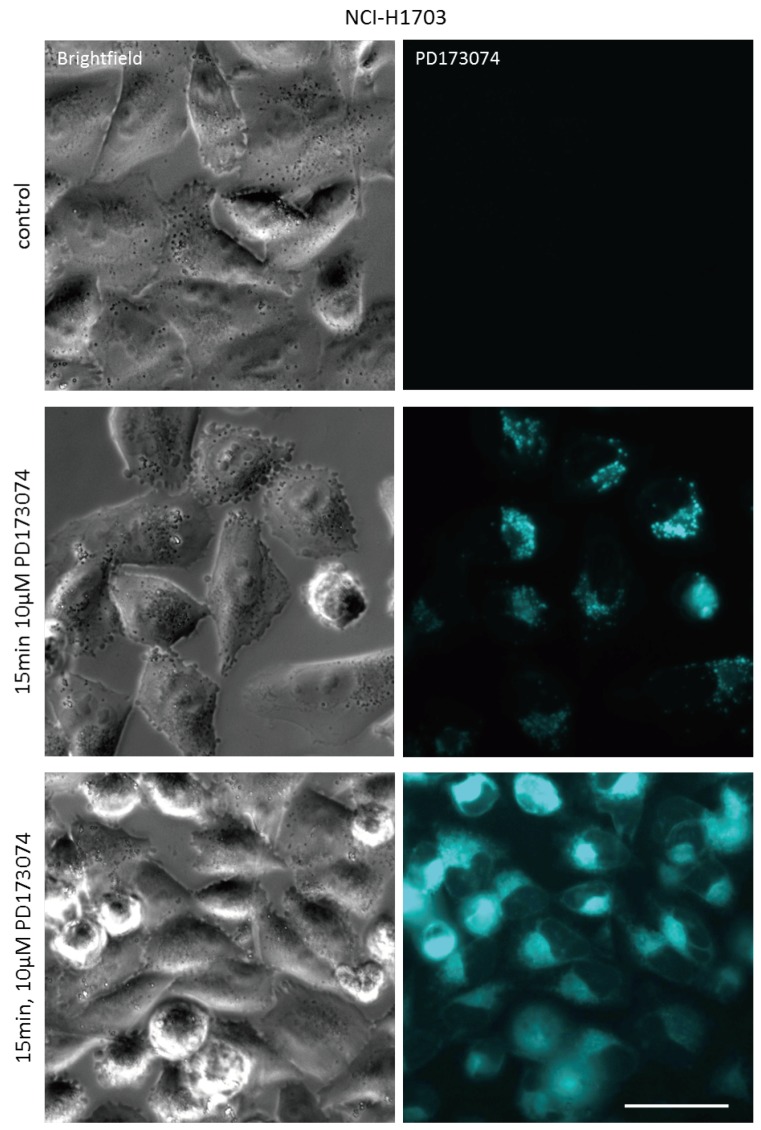
**Bafilomycin A1 pretreatment prevents lysosomal PD173074 sequestration.** Effect of 1 h preincubation with 1 µM bafilomycin A1 on subcellular distribution of PD173074 (10 µM) in NCI-H1703 cells was analyzed 15 min after drug exposure by live cell microscopy. The scale bar indicates 10 µm.

The identification of PD173074 as lysosomotropic agent suggested that compromising of lysosomal integrity might increase cytosolic drug levels, thereby augmenting the cytotoxic potential of PD173074. Chloroquine is an agent raising lysosomal pH by directly acting as luminal proton scavenger [33]. Indeed, coincubation with chloroquine altered the intracellular distribution of PD173074. As observed by live-cell microscopy, the addition of chloroquine to NCI-H1703 and NCI-H520 cells pretreated with the FGFR inhibitor converted the speckled lysosome-associated PD173074 signal to a more diffuse staining of the entire cytosolic space (Figure 3; Figure S3). Importantly, the same effect was observed upon coincubation with bafilomycin A1 (Figure 3; Figure S3). Bafilomycin A1 is a lysosomal vacuolar H^+^-ATPase (V-ATPase) inhibitor, specifically blocking H^+^-ion influx into lysosomes, and thereby inhibiting lysosomal acidification [34]. Analogously, pretreatment of NCI-H1703 and NCI-H520 cells with bafilomycin A1 for 1 h before addition of the FGFR inhibitor completely abolished lysosomal accumulation of the latter compound immediately after drug exposure (15 min; Figure 4; Figure S4).

### 3.4. Lysosomal alkalinization Enhances the Cytotoxic Potential of PD173074

**Figure 5:**
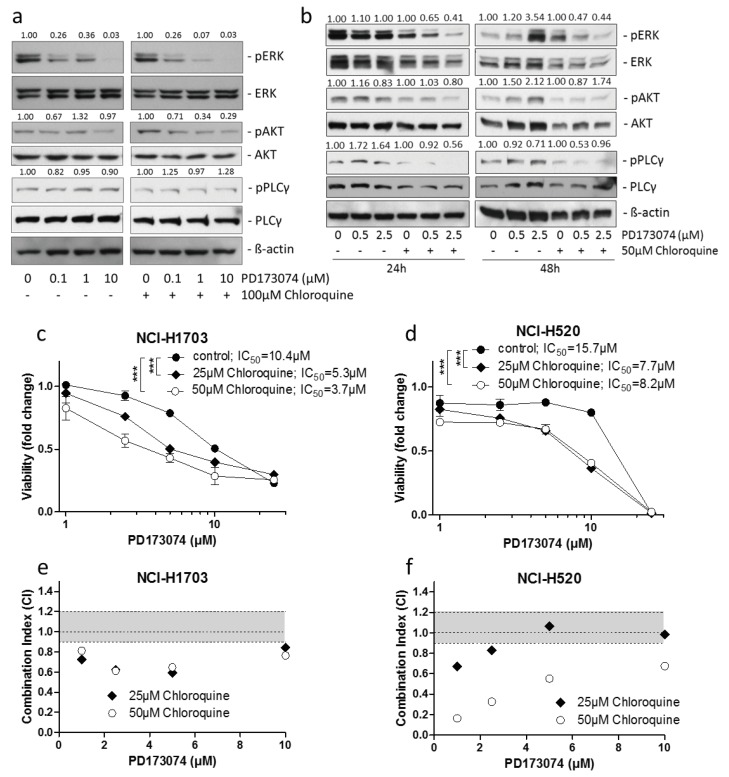
**Lysosome alkalinization sensitizes lung cancer cells towards PD173074.** (**a**) Impact of 100 µM chloroquine on the inhibitory potential of indicated PD173074 concentrations on FGFR signaling in NCI-H1703 cells, cotreated for 1 h was analyzed by Western blot analysis. Quantification of ERK, AKT, and PLCγ phosphorylation is shown. Values above respective lanes indicate the fold inhibition of ERK/AKT/PLCγ phosphorylation, are given normalized total ERK/AKT/PLCγ, respectively, as well as ß-actin expression levels and are shown relative to respective controls that were either untreated or treated only with chloroquine. ß-actin served as loading control. (**b**) Impact of 50 µM chloroquine on the inhibitory potential of increasing PD173074 concentrations on FGFR signaling in NCI-H1703 cells at the indicated time points was determined by Western blot analysis. Quantification of ERK, AKT, and PLCγ phosphorylation is shown. Values indicate the fold inhibition of ERK/AKT/PLCγ phosphorylation, are given normalized total ERK/AKT/PLCγ, respectively, and ß-actin expression levels and are shown relative to respective controls that were either untreated or treated only with chloroquine. ß-actin served as loading control. (**c**,**d**) Impact of 25 µM and 50 µM chloroquine on viability of NCI-H1703 (**c**) and NCI-H520 (**d**) cells cotreated for 72 h with rising concentrations of PD173074 was analyzed by MTT assay. *** *p* < 0.001, two-way ANOVA, Bonferroni post-test. (**e**,**f**) Synergism of PD173074 and chloroquine in NCI-H1703 (**e**) and NCI-H520 (**f**) cells was evaluated calculating CalcuSyn combination indices (CI). CI values above 1.2, between 0.9–1.1 and below 0.9 indicated antagonism, additivity, and synergism, respectively.

Consequently, it was of interest whether the observed shift in intracellular distribution of PD173074 might impact on its anticancer activity. Of note, raising the lysosomal pH resulted in a moderately decreased overall intracellular PD173074 fluorescence intensity (Figure S5A). Repetition of cell-free 3D fluorescence spectroscopy revealed that in the used concentration bafilomycin A1 was non-fluorescent (data not shown). Chloroquine emitted fluorescence with excitation and emission peaks at 330 nm and 384 nm, respectively, while in the wavelength range relevant for PD173074 fluorescence, very low activity was detected (Figure S5B). Accordingly, neither bafilomycin A1 nor chloroquine induced any spectral shift or altered intensity of PD173074 fluorescence (Figure S5C,D). Western blot analysis of PD173074-treated NCI-H1703 cells coincubated with chloroquine for 1 h revealed a distinctly enhanced inhibitory potential on FGFR downstream signaling, especially in case of the mitogen-activated protein kinase (MAPK) pathway. At least in case of 1 µM PD173074, this was illustrated by increased suppression of ERK phosphorylation in cotreated samples as compared to cells exposed to the FGFR inhibitor only (14.29-fold versus 2.78-fold inhibition, respectively) (Figure 5A). In case of 1 µM and 10 µM PD173074, enhanced inhibition in the presence of chloroquine was also apparent for the AKT/PKB pathway (2.94-fold versus 0.76-fold inhibition at 1 µM, and 3.45-fold versus 1.03-fold, respectively). In contrast, activity of the PLCγ pathway was virtually unaffected by PD173074 treatment irrespective of the presence or absence of chloroquine. In a different experimental setting, treatment of NCI-H1703 cells with PD173074 for up to 48 h revealed that inhibition of FGFR downstream signaling was markedly sustained in case of coincubation with chloroquine (Figure 5B). Here, it is noteworthy that the inhibitory potential of PD173074 as single agent on ERK phosphorylation was completely lost after 24 h (0.91-fold and 1.00-fold inhibition at 0.5 and 2.5 µM PD173074, respectively). After 48 h, even hyperactivation of this signaling arm was apparent in case of 2.5 µM drug exposure (0.83-fold and 0.28-fold inhibition at 0.5 and 2.5 µM PD173074, respectively), potentially reflecting a positive feedback activation loop. In contrast, in cells concomitantly exposed to chloroquine, inhibition of ERK phosphorylation was sustained over time (1.54-/2.44-fold (24 h) and 2.13-/2.27-fold (48 h) inhibition at 0.5 and 2.5 µM PD173074, respectively). Importantly, in this experimental setting (at both 24 h and 48 h), inhibition of the AKT/PKB and PLCγ pathways by PD173074 was more efficient in the presence of chloroquine at virtually all tested concentrations. These data suggest that lysosomal alkalinization suppresses lysosomal PD173074 sequestration, thus elevating its active concentration at the critical FGFR target site at the cytoplasmic face of the plasma membrane. Consequently, we analyzed whether compromising of lysosomal integrity also may increase the sensitivity of cancer cells towards PD173074. Indeed, coincubation of NCI-H1703 and NCI-H520 cells with chloroquine and bafilomycin A1 significantly increased the cytotoxic potential of PD173074, resulting in distinctly additive, but mostly even synergistic toxic effects (Figure 5C–F; Figure S5E–H). It needs to be mentioned that - while bafilomyicin A1 concentrations were applied at subtoxic concentrations - for chloroquine, incubation of cells at 50 µM moderately reduced cell viability as single agent, whereas it proved to be subtoxic at lower concentrations for both cell lines (Figure S5I,J). These synergistic effects resulted in case of chloroquine in 2.8-fold and 1.9-fold reduced IC_50_ values of PD173074 in case of NCI-H1703 and NCI-H520 cells, respectively. For 10 nM Bafilomycin A1, a similarly increased activity of PD173074 was observed, albeit to a slightly lesser extent as compared to chloroquine (1.9-fold and 1.3-fold reduced IC_50_ values for NCI-H1703 and NCI-H520 cells, respectively). The fact that only little increases in synergism were observed upon raising the levels of either lysosomal inhibitor indicates a saturation effect of lysosomal alkalinization, above which PD173074 accumulation is not reversible any further. Taken together, these findings imply a role of lysosomal sequestration as the critical negative determinant of the PD173074 anticancer potential.

## 4. Discussion

In biomedical research, modes-of-action of modern targeted compounds are generally predicted by complex computer-assisted in silico modeling, followed by cell-biological validation and high-resolution imaging procedures to visualize compound interaction with respective molecular targets [35,36,37,38]. However, an additional factor impacting on compound efficacy is defined by its intracellular behavior. Knowledge of the intracellular dynamics of anticancer compounds is crucial, as it becomes increasingly evident that - besides known adaptive mechanisms such as target mutation, activation of alternative oncogenic pathways or transmembrane-transporter-mediated drug efflux - also other factors such as selective compound sequestration to cellular organelles have an impact on compound efficacy and can result in cellular unresponsiveness [20,21,39]. In general, knowledge of intracellular pharmacokinetics is limited due to the highly complex nature of technologies capable of resolving compounds at subcellular level. These include elegant technologies such as hyperspectral Raman scattering spectroscopy [30], nanoscale secondary ion mass spectrometry (nanoSIMS [40]) or diaminobenzidine (DAB) photo-oxidation [41]. The latter two approaches combine ultrastructural resolution methods with analytical chemistry. However, these technologies are restricted to the detection of metal-containing compounds (nanoSIMS) or to the physicochemical ability of compounds to generate reactive oxygen species upon excitation with light of specific wavelengths (DAB photo-oxidation [41,42]). Alternatively, artificial drug labeling represents a feasible strategy for intracellular tracking which, however, bears the risk of altering the pharmacological behavior of the respective compound [43]. Intrinsic fluorescence activity has been described for only a limited number of chemotherapeutic and targeted agents including doxorubicin, mitoxantrone, topotecan, gefitinib, erlotinib, sunitinib and nintedanib [16,17,18,19,20]. Nevertheless, exploitation of these fluorescence properties to study the intracellular compound dynamics has generated substantial advancements in the understanding of their modes of action and - importantly - also their limitations and failure [19,23,30,39]. As such, ion-based sequestration of widely used therapeutic agents such as doxorubicin, mitoxantrone, sunitinib, but also the FGFR inhibitor nintedanib to lysosomes was identified as a cancer cell defense mechanism [16,19,20,44]. These means to reduce cytoplasmic levels of pharmacologic agents was described to be further fostered by increased transcription factor EB (TFEB)-orchestrated lysosomal biogenesis and - as shown for topotecan, sunitinib and doxorubicin - by exocytosis of intralysosomal contents [22]. At least in the case of nintedanib, which is clinically approved for the treatment of non-small cell lung cancer, concomitant targeting of lysosomes was shown to potentiate its activity against lung cancer cell lines in vitro [20]. This goes well in accordance with the here-presented data for PD173074, especially because nintedanib is also considered a lipophilic (logP at pH 7.4 = 3.0), weakly basic compound, with a pKa of 7.9, based on the presence of a piperazine moiety exhibiting the potential for protonation. As mentioned above, another clinically approved tyrosine kinase inhibitor, imatinib, has been shown to accumulate in lysosomes [30]. Also in this case, a pKa (8.1) comparable to that of nintedanib (due to the presence of a piperazine moiety in both molecules) reflects compound properties making lysosomotropism likely. It is tempting to ask whether chemical modification of anticancer compounds to reduce lysosomotropism might enhance their cytotoxic potential. Extensive research efforts would be necessary to identify derivatives exhibiting optimized intracellular pharmacokinetics, reduced organelle sequestration and, at the same time, sustained anticancer activity. This might prove challenging in the light of the fact that minor modifications in the chemical make-up of a compound are likely to strongly affect its physicochemical properties (including lipophilicity and the tendency to become protonated) and, thus, its pharmacological behavior. Consequently, it is conceivable that optimization of therapy containing lysosomotropic agents may best be achieved by rationale combination with agents targeting lysosomal integrity. Attempts have been launched to compromise the lysosomal integrity of cancer cells in order to increase the cytotoxic potential of lysosomotropic anticancer compounds. As mentioned, such approaches include pharmacological lysosome alkalinization via luminal proton scavenging (e.g., by chloroquine) or v-ATPase inhibition (e.g., by bafilomycin A1) [45]. Other strategies employ pharmacological Trojan horses such as imidazoacridinones or so-called photoaccoustic nanobombs, which themselves exhibit lysosomotropic properties. These have been developed for the photodynamic destruction of lysosomes to induce cancer cell death and might prove beneficial for combination with lysosomotropic anticancer compounds aimed at other cellular targets [46,47,48].

In addition to lysosomes, other organelles are implicated in the scavenging of pharmacological agents. For instance, adiposomal accumulation of the preclinical compound curcumin was suggested to lower its anticancer activity [49]. Another work described late endosomal sequestration of doxorubicin to mediate cancer cell resistance [23]. Accordingly, these studies demonstrated that combination treatments with compounds compromising the physiological integrity of the above-mentioned cellular compartments yield synergistic anti-proliferative effects, presumably by elevating drug levels available to act at their respective target sites. In line with these observations, in our study we have identified lysosomal release and enhanced toxicity in FGFR-driven lung cancer cells by combination of PD173074 with compounds targeting lysosomal pH. This is most likely mediated by enhanced local drug concentrations at the intracellular side of the membrane to interact with the FGFR kinase domain.

## 5. Conclusions

Taken together, the intrinsic fluorescence properties of PD173074–identified in this study–provide a tool to facilitate studies on the intracellular dynamics and cytotoxic activity of this FGFR inhibitor. Deeper insights into the subcellular distribution of this drug might form the basis for enhancing its therapeutic efficacy, e.g., by rationale combination regimens with lysosome-targeting agents, such as chloroquine, which is already widely used in the clinics for antimalarial therapy. As a consequence, potentiated anticancer activity of PD173074 might generate the opportunity for dose reduction to improve systemic tolerability in patients, which currently constitutes a major limitation to the clinical applicability of this compound.

## Figures and Tables

**Table 1:** Fluorescence properties of intracellular PD173074.

Cell Line	Laser (nM)	Bandpass Filter (nm)	Fluorescence Intensity (a.u. mean ± SD) ^a^		Relative Fluorescence Increase ^b^
			Control	PD173074	
NCI-H1703	405	450/40	7.99 ± 0.08	4129.54 ± 43.02	516.8 ^***^
NCI-H520	405	450/40	11.34 ± 0.10	6210.24 ± 191.27	547.6 ^***^

^a^ Cells were exposed to 10 µM PD173074 for 1 h. Fluorescence intensity was analyzed by flow cytometry; ^b^ Fluorescence increase of drug-treated cells is shown relative to untreated controls; *** *p* < 0.001; student’s *t*-test. a.u., arbitrary units.

## Supplementary Materials

The following are available online at http://www.mdpi.com/2073-4409/7/12/259/s1, Figure S1: Fluorescence-based in vitro and cell-free detection of PD173074, Figure S2: Lysosomal localization of PD173074 remains stable over several days, Figure S3: Lysosomal de-acidification reduces PD173074 accumulation in lysosomes, Figure S4: Bafilomycin A1 pretreatment prevents lysosomal PD173074 sequestration, Figure S5: Lysosomal alkalinization increases the cytotoxic potential of PD173074.
