# Supplementary material for: Lysosomal Sequestration Impairs the Activity of the Preclinical FGFR Inhibitor PD173074
Source: Cells. 2018 Dec 8;7(12):259. doi: 10.3390/cells7120259 (PMC6315953; doi:10.3390/cells7120259)
Supplement: Supplementary file 1 [file cells-07-00259-s001.pdf]

Supplementary figures and figure legends

Figure S5

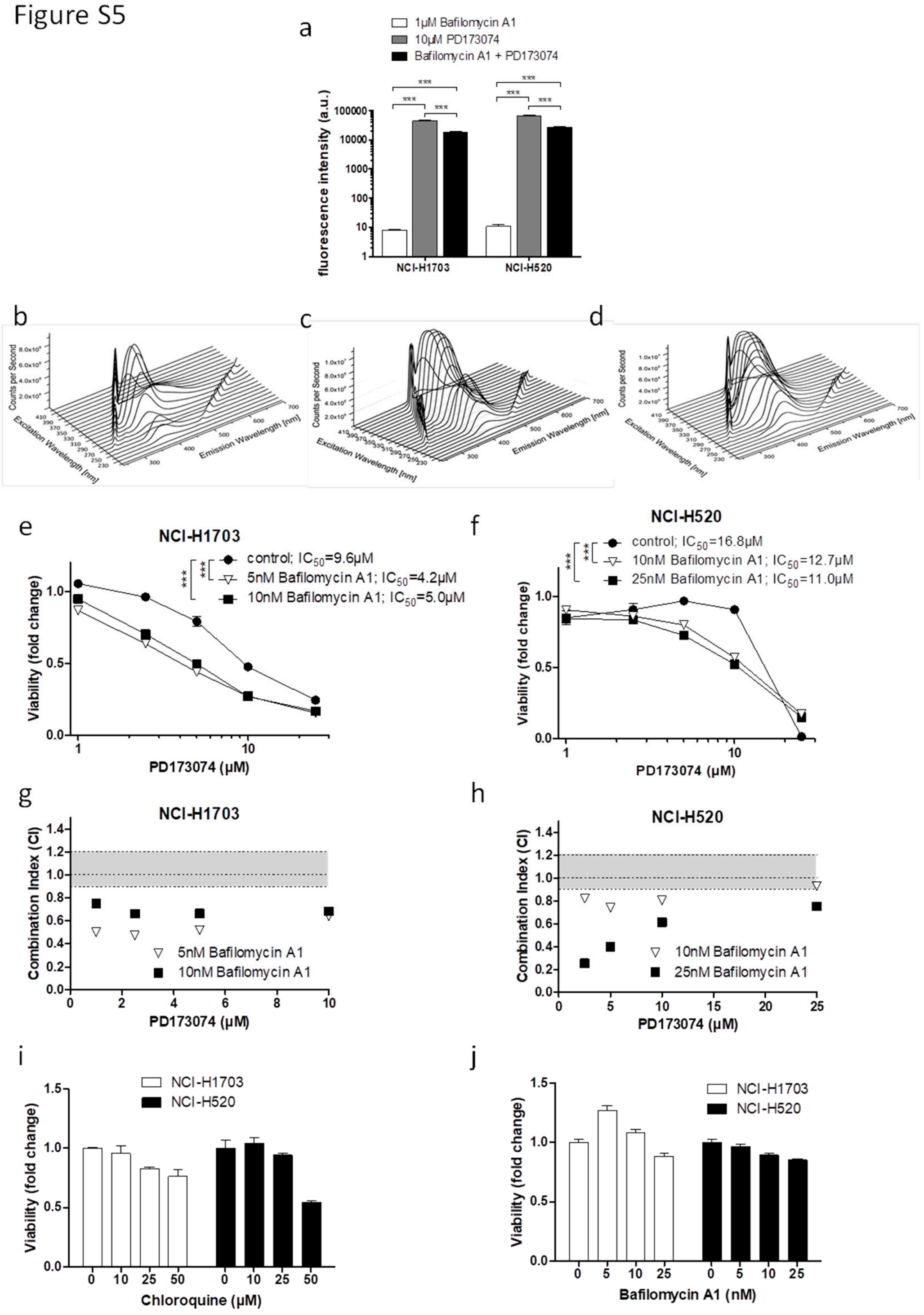

Figure S1. Fluorescence-based *in vitro* and cell-free detection of PD173074. (a) Intracellular fluorescence of NCI-H520 cells, treated for 1 h with 10 µM PD173074 was determined by flow cytometry. Fluorescence emission was detected using DAPI (450/40 nm), Horizon V450 (450/40 nm), FITC (530/30 nm) and APC (660/20 nm) emission channels for the 355 nm, 405 nm, 488 and 640 nm lasers, respectively. ***$p < 0.001$, student's t-test. **(b)** Accumulation of indicated PD173074 concentrations in NCI-H520 cells was measured over time by flow cytometry using the 405 nm laser and the Horizon V450 (450/40 nm) emission filter. ***$p < 0.001$, two-way ANOVA, Bonferroni post-test. **(c)** A 3-dimensional, full-range excitation-emission spectrum was generated by fluorescence spectroscopy to analyze cell-free fluorescence properties of PD173074 at pH 4,5,6 (diluted in 1% DMSO/citrate buffer) and 7.4 (diluted in 1% DMSO/PBS). Excitation wavelengths ranged from 220 nm to 420 nm, emission spectra were recorded from 250 nm to 700 nm. Diagonal ridges indicate Raleigh scatters of first and second order. **(d)** Intracellular PD173074 distribution in VL-2 and BEAS-2B cells, exposed to 10 µM of the drug for 1 h was investigated by confocal microscopy using the DAPI channel. Lysosomes were stained with LysoTracker Red®. The scale bar indicates 10 µm. **(e)** Accumulation of indicated PD173074 concentrations in NCI-H1703 cells preincubated–or not–with 20 µM CPZ was measured over time by flow cytometry using the 405 nm laser and the Horizon V450 (450/40 nm) emission filter. ***$p < 0.001$, two-way ANOVA, Bonferroni post-test. ns, non-significant;

Figure S2

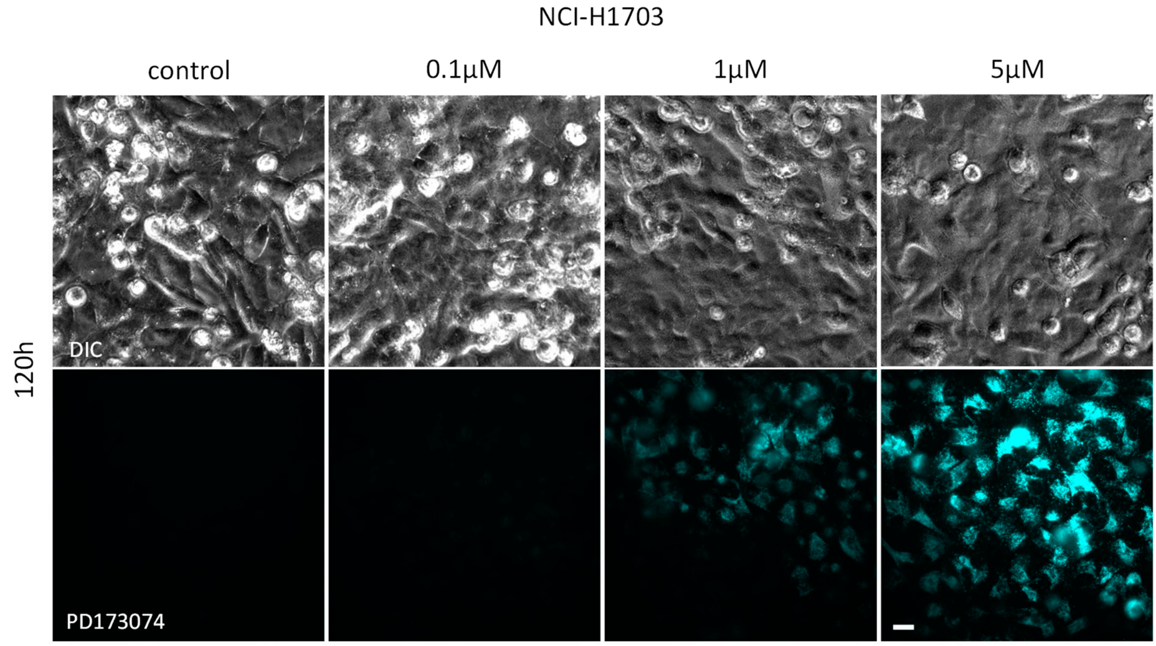

Figure S2. Lysosomal localization of PD173074 remains stable over several days. PD173074 retention in lysosomes of NCI-H1703 cells was analyzed by live cell microscopy. Cells were treated for 1 h with indicated concentrations of PD173074, followed by incubation in drug-free media. PD173074 was imaged using the DAPI channel and is pseudocolored in cyan. The scale bar indicates 10 μm.

Figure S3

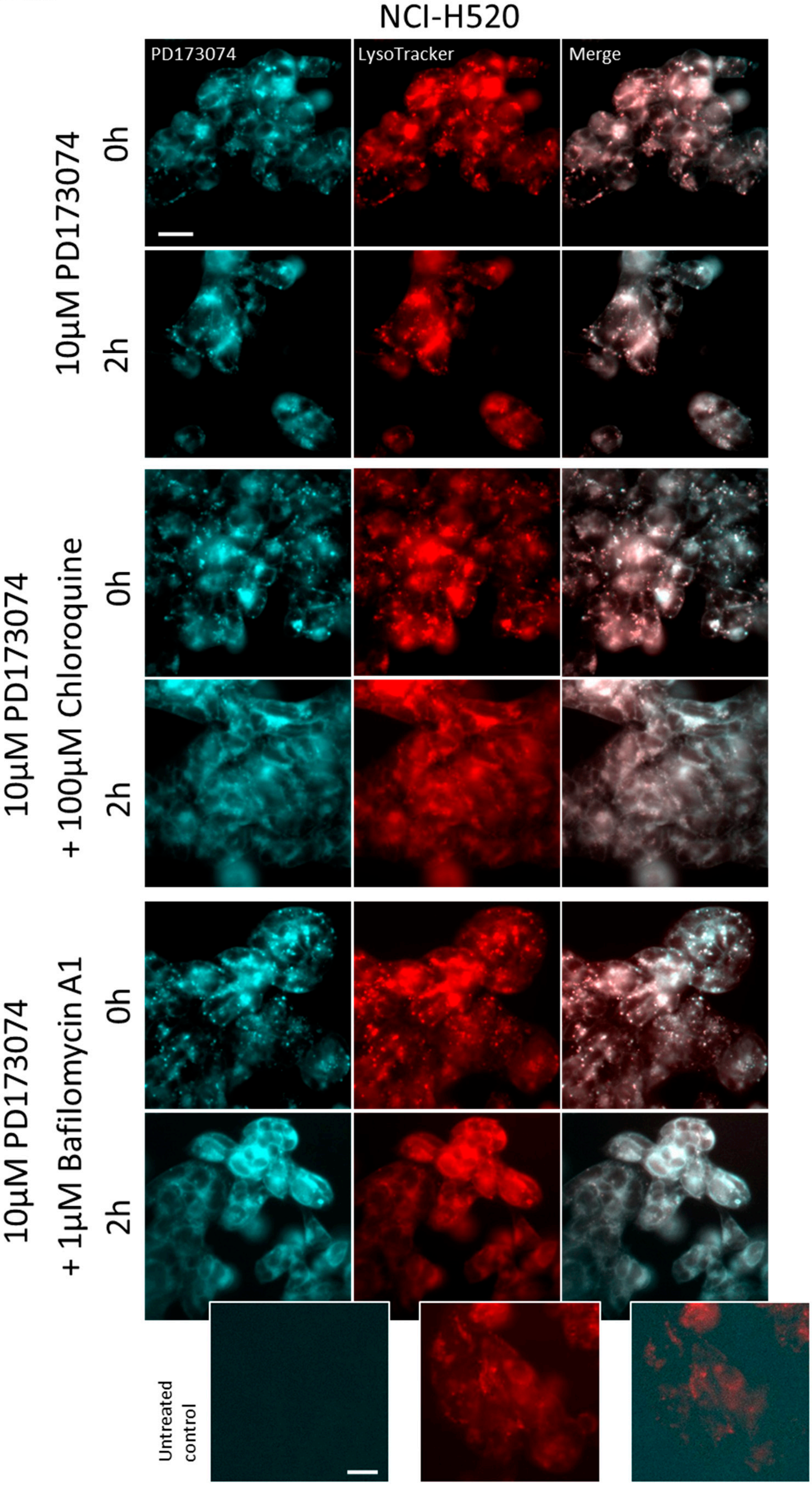
NCI-H520
PD173074
LysoTracker
Merge
10µM PD173074
0h
2h
10µM PD173074
+ 100µM Chloroquine
0h
2h
10µM PD173074
+ 1µM Bafilomycin A1
0h
2h
Untreated
control

Figure S3. Lysosomal de-acidification reduces PD173074 accumulation in lysosomes. Effect of 2 h coincubation with 100 μM chloroquine or with 1 μM bafilomycin A1 on subcellular distribution of PD173074 (10 μM) in NCI-H520 cells was analyzed by live cell microscopy. LysoTracker Red® was used to stain lysosomes. The scale bar indicates 10 μm.

Figure S4

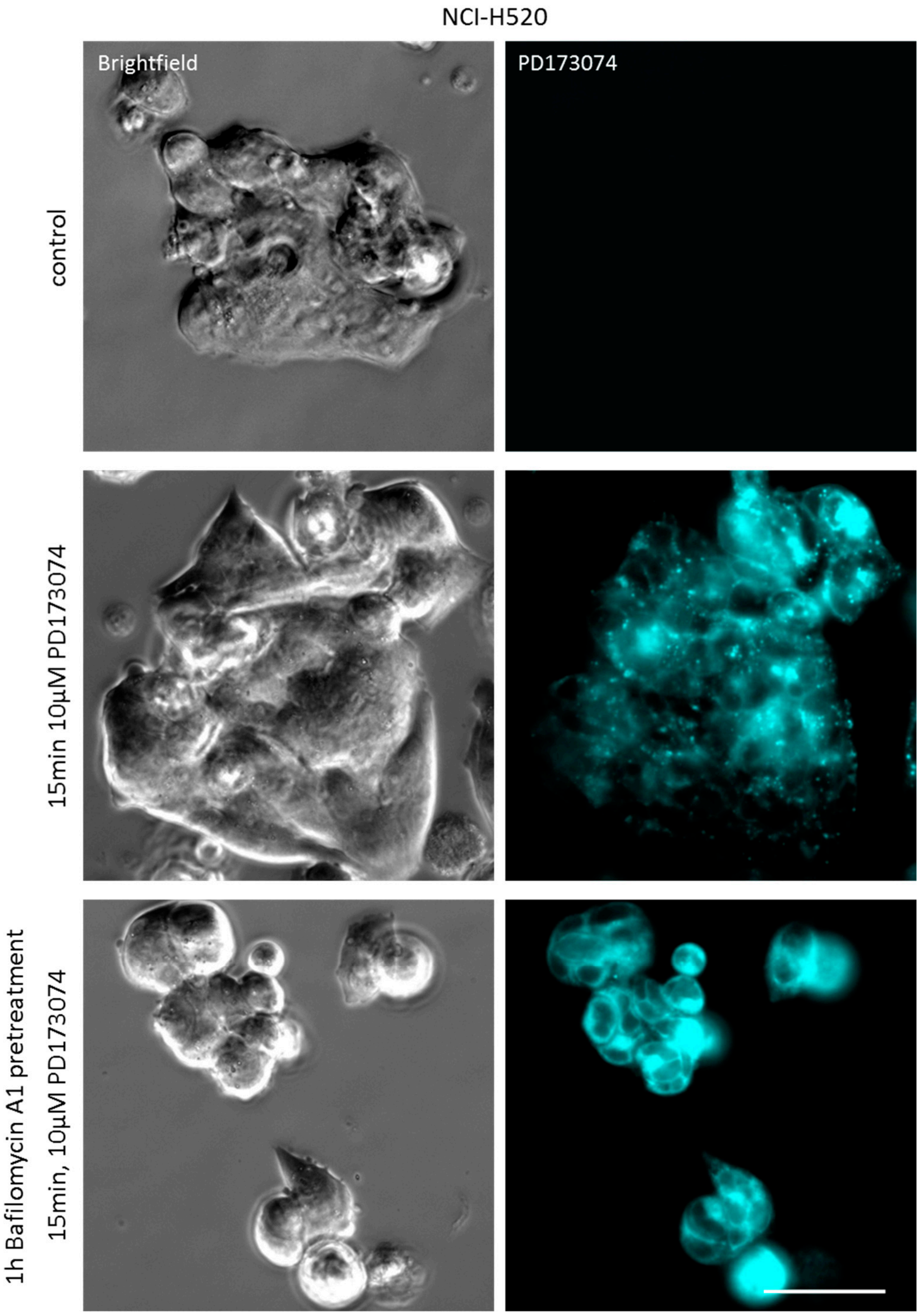

Figure S4. Bafilomycin A1 pretreatment prevents lysosomal PD173074 sequestration. Effect of 1 h preincubation with 1 µM bafilomycin A1 on subcellular distribution of PD173074 (10 µM) in NCI-H520 cells was analyzed 15 min after drug exposure by live cell microscopy. The scale bar indicates 10 µm.

Figure S5

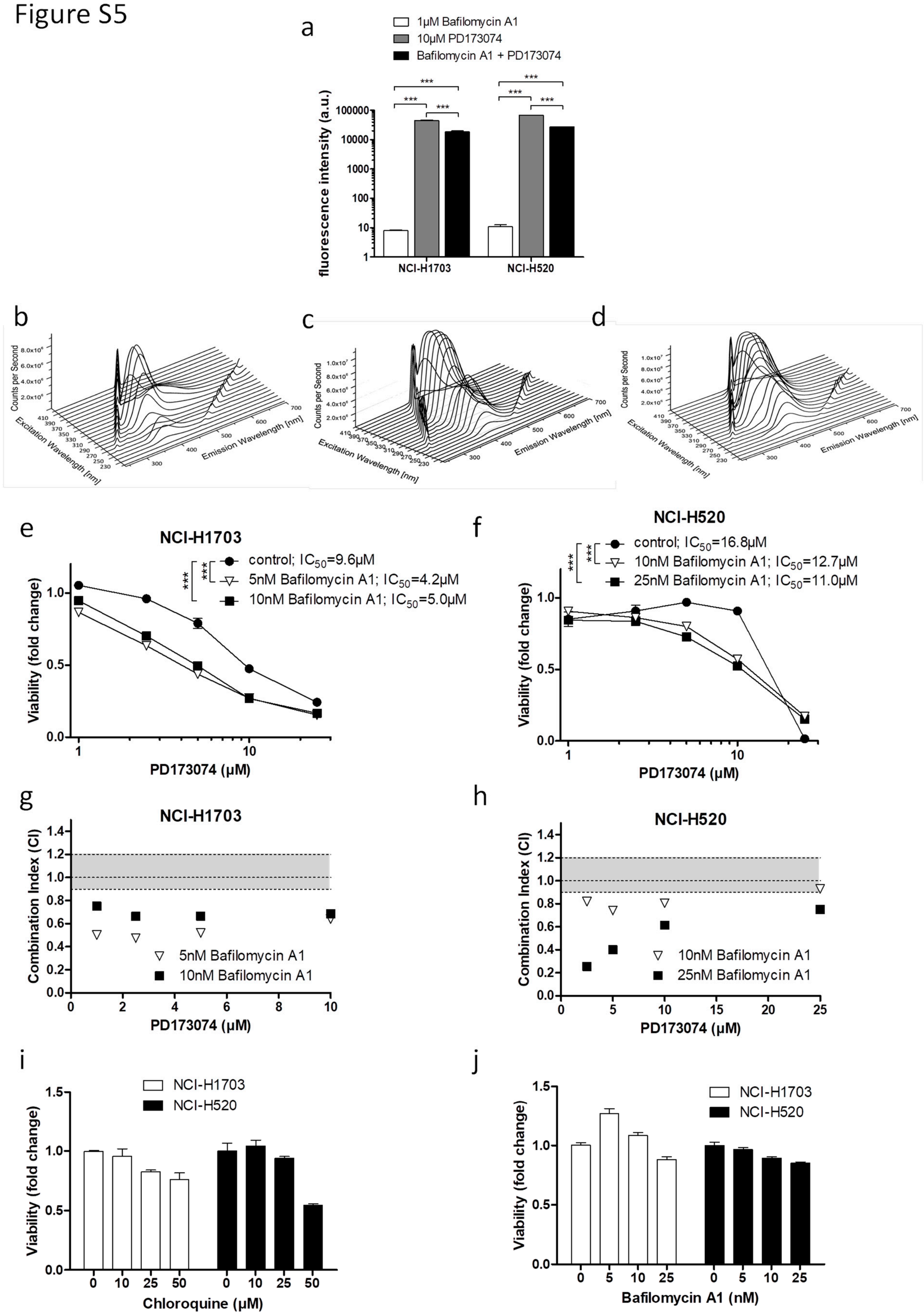
a
1µM Bafilomycin A1
10µM PD173074
Bafilomycin A1 + PD173074
***
fluorescence intensity (a.u.)
100000
10000
1000
100
10
1
NCI-H1703
NCI-H520
b
c
d
Counts per Second
Excitation Wavelength [nm]
Emission Wavelength [nm]
e
NCI-H1703
control; IC50=9.6µM
5nM Bafilomycin A1; IC50=4.2µM
10nM Bafilomycin A1; IC50=5.0µM
Viability (fold change)
PD173074 (µM)
f
NCI-H520
control; IC50=16.8µM
10nM Bafilomycin A1; IC50=12.7µM
25nM Bafilomycin A1; IC50=11.0µM
Viability (fold change)
PD173074 (µM)
g
NCI-H1703
Combination Index (CI)
5nM Bafilomycin A1
10nM Bafilomycin A1
PD173074 (µM)
h
NCI-H520
Combination Index (CI)
10nM Bafilomycin A1
25nM Bafilomycin A1
PD173074 (µM)
i
NCI-H1703
NCI-H520
Viability (fold change)
Chloroquine (µM)
j
NCI-H1703
NCI-H520
Viability (fold change)
Bafilomycin A1 (nM)

Figure S5. Lysosomal alkalinization increases the cytotoxic potential of PD173074. (a). Impact of 1 h coincubation of 1 μM bafilomycin A1 on intracellular accumulation of PD173074 (10 μM) in NCI-H1703 and NCI-H520 cells was determined by flow cytometry using the 405 nm laser and the Horizon V450 (450/40 nm) emission filter. ***$p<0.001$, two-way ANOVA, Bonferroni post-test. **(b-d)** A 3-dimensional, full-range excitation-emission spectrum was generated by fluorescence spectroscopy to analyze cell-free fluorescence properties of 15 μM chloroquine (b) and combination of 15 μM PD173074 in combination with 1 μM bafilomycin A1 (c) or 15 μM chloroquine (d) at pH 7.4 (diluted in 1% DMSO/PBS). Excitation wavelengths ranged from 220 nm to 420 nm, emission spectra were recorded from 250 nm to700 nm. Diagonal ridges indicate Raleigh scatters of first and second order. **(e)** Impact of 5 nM and 10 nM bafilomycin A1 on viability of NCI-H1703 cells cotreated for 72 h with rising concentrations of PD173074 was determined by MTT assay. ***$p<0.001$, two-way ANOVA, Bonferroni post-test. **(f)** Impact of 10 nM and 25 nM bafilomycin A1 on viability of NCI-H520 cells cotreated for 72 h with rising concentrations of PD173074 was determined by MTT assay. ***$p<0.001$, two-way ANOVA, Bonferroni post-test. **(g, h)** Synergism of PD173074 and bafilomycin A1 in NCI-H1703 (g) and NCI-H520 (h) cells was evaluated calculating CalcuSyn combination indices (CI). CI values above 1.2, between 0.9 - 1.2 and below 0.9 indicated antagonism, additivity, and synergism, respectively. **(i)** Impact of indicated concentrations of chloroquine on viability of NCI-H1703 and NCI-H520 cells was determined by MTT viability assay after 72 h drug exposure. **(j)** Impact of indicated concentrations of bafilomycin A1 on viability of NCI-H1703 and NCI-H520 cells was determined by MTT viability assay after 72 h drug exposure.
